# Supplementary material for: KRAS and NRAS Translation Is Increased upon MEK Inhibitors-Induced Processing Bodies Dissolution
Source: Cancers (Basel). 2023 Jun 6;15(12):3078. doi: 10.3390/cancers15123078 (PMC10296394; doi:10.3390/cancers15123078)
Supplement: Supplementary file 1 [file cancers-15-03078-s001.zip › Figure S1.pdf]

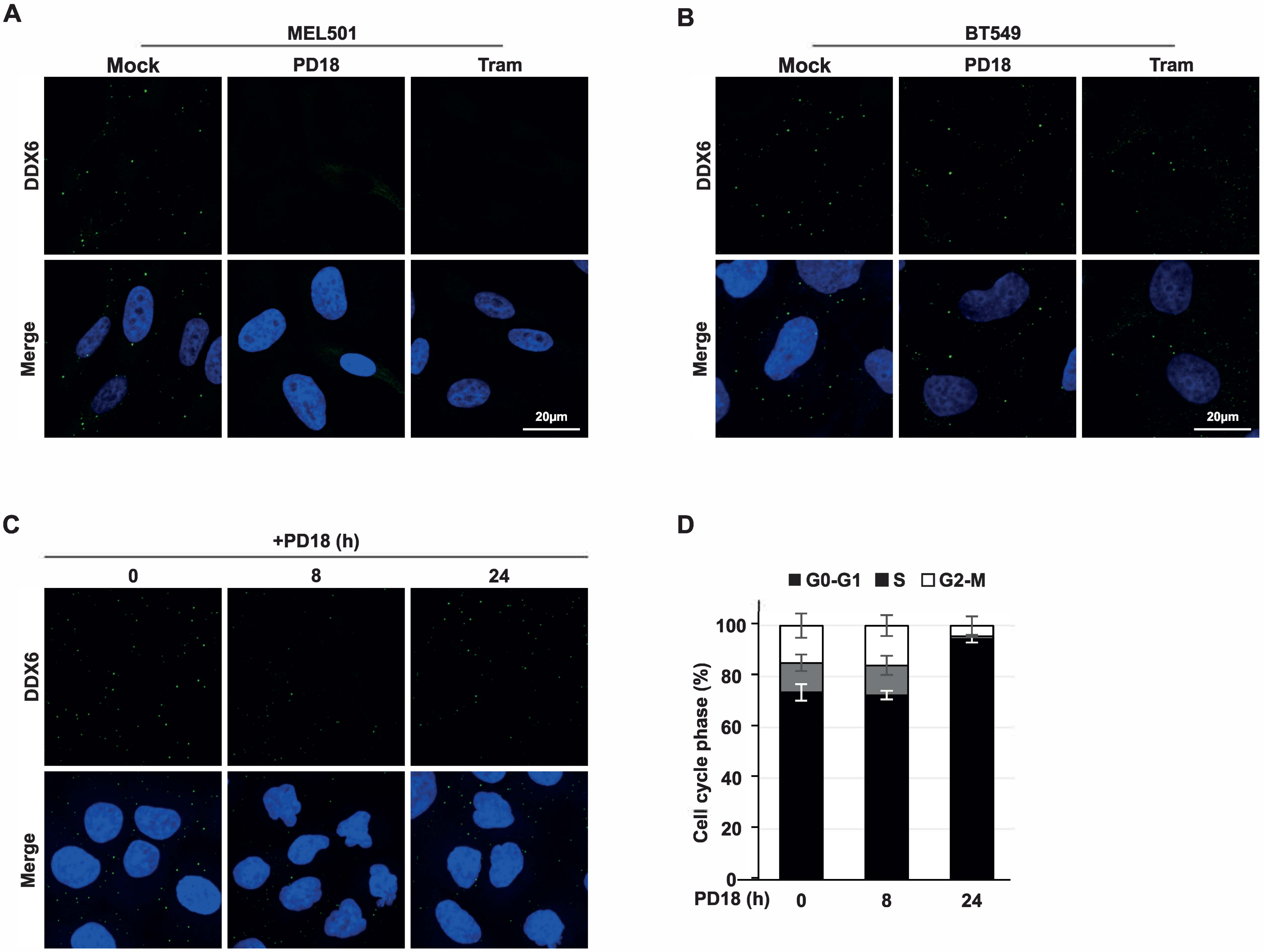

Supplementary Figure S1: Effect of MEKi on Processing bodies and cell cycle.
A. MEL501 and **B.** BT549 cells were treated 24h with PD184352 (PD18) or trametinib (Tram). **C.D.** A549 cells were treated with PD184352 (PD18) at 10µM and harvested at indicated time. **A-C.** Confocal analysis of P-body using anti-DDX6 antibodies (Green) with DAPI nuclear staining (Blue). **D.** Cell cycle distribution was analyzed by flow cytometry. Results represent the merge of 3 independent experiments.

Supp. Figure S1
