# Supplementary material for: KRAS and NRAS Translation Is Increased upon MEK Inhibitors-Induced Processing Bodies Dissolution
Source: Cancers (Basel). 2023 Jun 6;15(12):3078. doi: 10.3390/cancers15123078 (PMC10296394; doi:10.3390/cancers15123078)
Supplement: Supplementary file 1 [file cancers-15-03078-s001.zip › Figure S2.pdf]

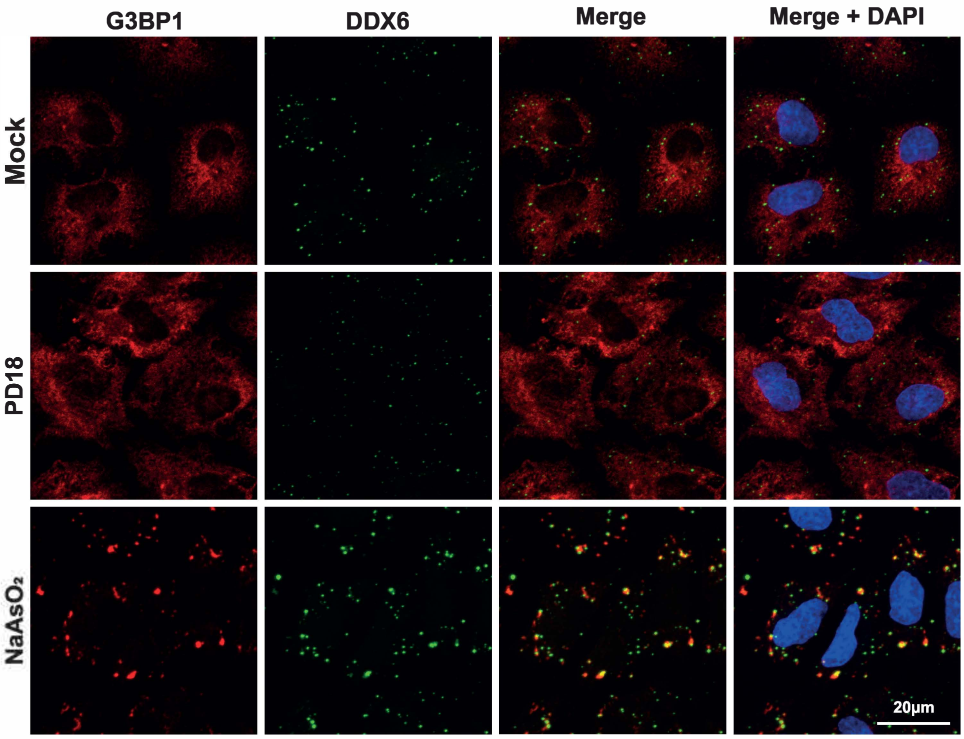

Supplementary Figure S2: MEK inhibitors do not induce stress granule formation.`
A549 cells were treated 24h with PD184352 (PD18) at 10µM or 20min with Sodium Arsenite (NaAsO2) at 0.5mM. Confocal analysis of P-body using anti-DDX6 (Green) and stress granule anti-G3BP1 (Red) antibodies respectively with DAPI nuclear staining (Blue).

Supp. Figure S2
