# Supplementary material for: KRAS and NRAS Translation Is Increased upon MEK Inhibitors-Induced Processing Bodies Dissolution
Source: Cancers (Basel). 2023 Jun 6;15(12):3078. doi: 10.3390/cancers15123078 (PMC10296394; doi:10.3390/cancers15123078)
Supplement: Supplementary file 1 [file cancers-15-03078-s001.zip › Figure S3.pdf]

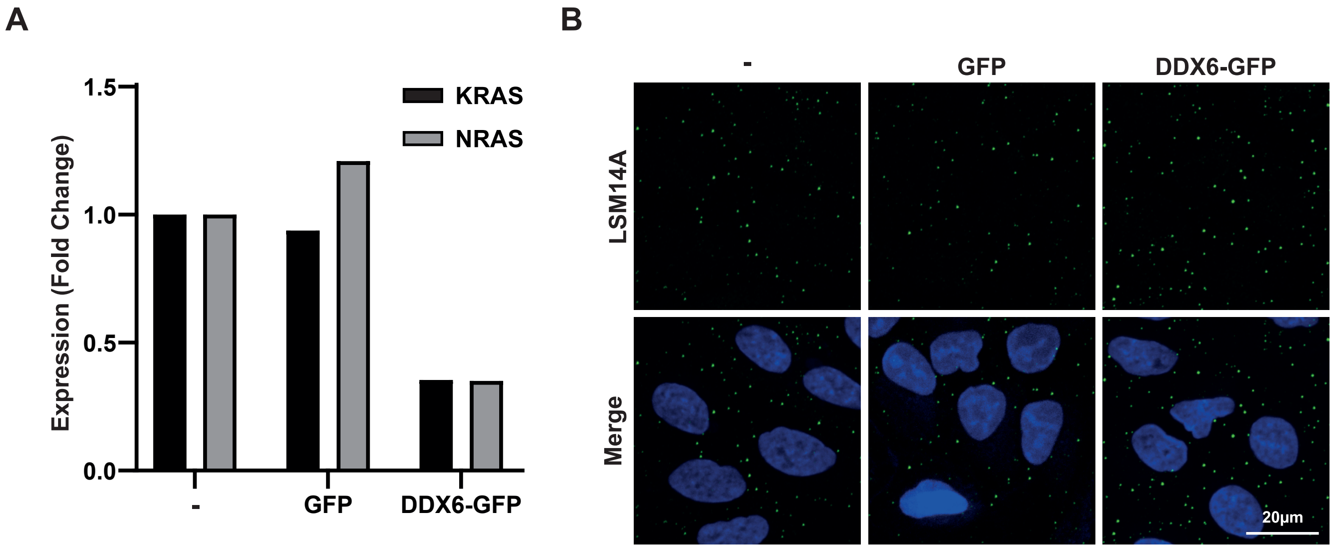

Supplementary Figure S3: DDX6 overexpression increase P-body number and decrease KRAS and NRAS translation.

A-B. A549 wild type (-) cells, overexpressing a Green fluorescent protein (GFP) or a DDX6-GFP fused protein (DDX6). **A.** Western blot quantification of KRAS and NRAS at the indicated time. Results represent the merge of 2 independent experiments. **B.** Confocal analysis of P-body using anti-LSM14A (Green) antibodies with DAPI nuclear staining (Blue).

Supp. Figure S3
