# Supplementary material for: KRAS and NRAS Translation Is Increased upon MEK Inhibitors-Induced Processing Bodies Dissolution
Source: Cancers (Basel). 2023 Jun 6;15(12):3078. doi: 10.3390/cancers15123078 (PMC10296394; doi:10.3390/cancers15123078)
Supplement: Supplementary file 1 [file cancers-15-03078-s001.zip › Figure S4.pdf]

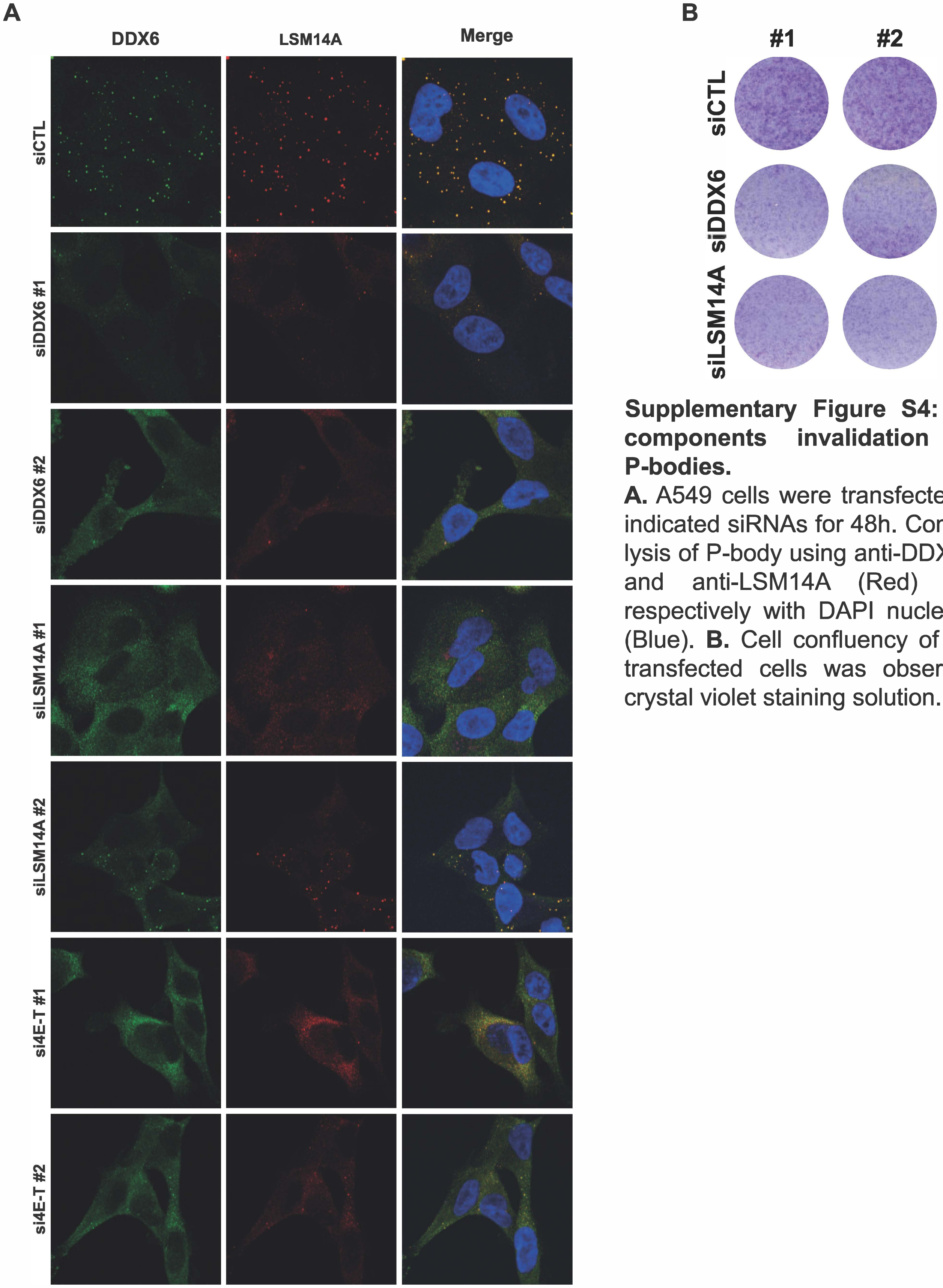

Supplementary Figure S4: P-bodies components invalidation dissolve P-bodies.

A. A549 cells were transfected with the indicated siRNAs for 48h. Confocal analysis of P-body using anti-DDX6 (Green) and anti-LSM14A (Red) antibodies respectively with DAPI nuclear staning (Blue). **B.** Cell confluency of remaining transfected cells was observed using crystal violet staining solution.

Supp. Figure S4
