# Supplementary material for: KRAS and NRAS Translation Is Increased upon MEK Inhibitors-Induced Processing Bodies Dissolution
Source: Cancers (Basel). 2023 Jun 6;15(12):3078. doi: 10.3390/cancers15123078 (PMC10296394; doi:10.3390/cancers15123078)
Supplement: Supplementary file 1 [file cancers-15-03078-s001.zip › Figure S5.pdf]

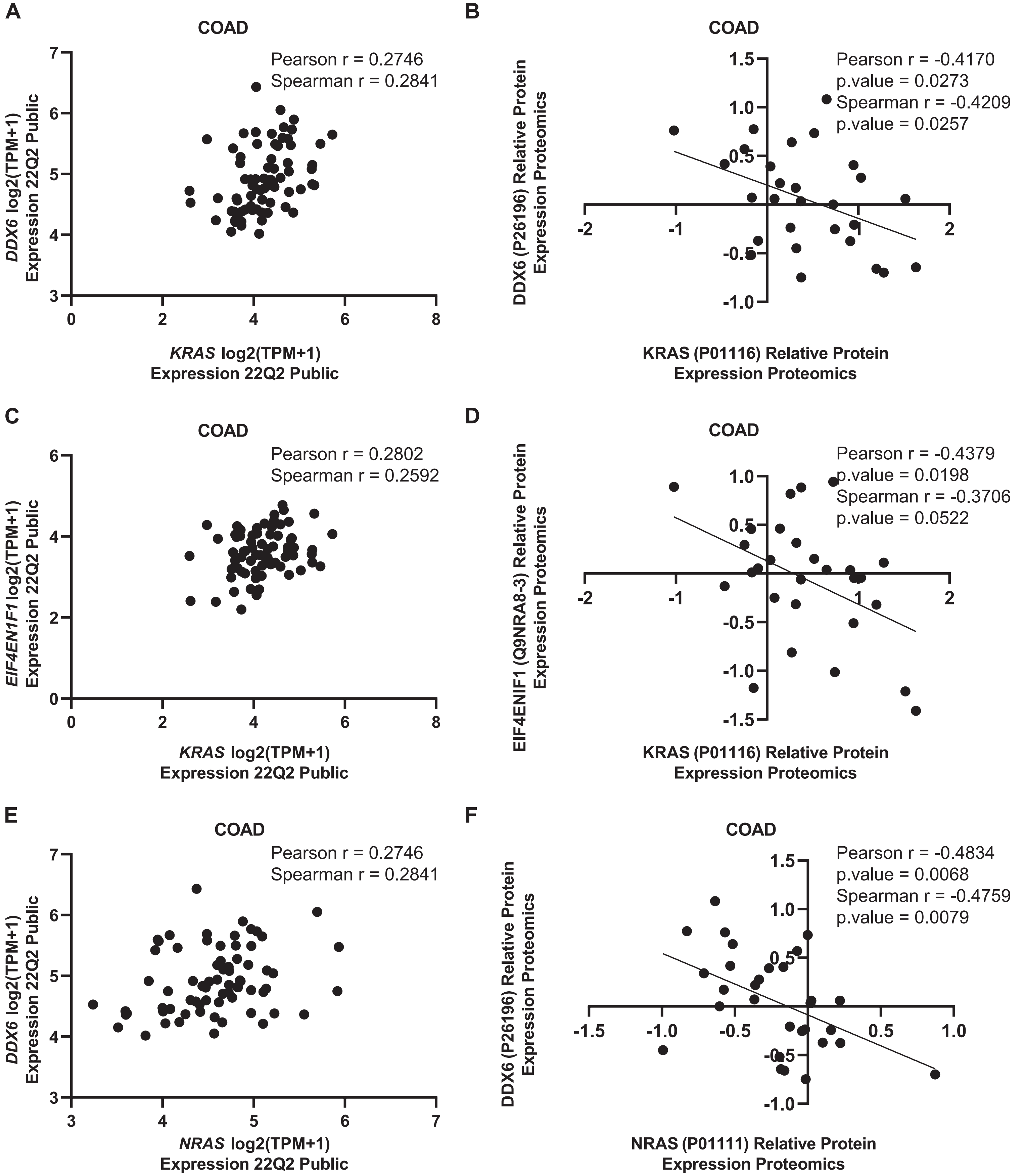

Supplementary Figure S5: P-body protein levels inversely correlate with KRAS and NRAS expression levels in Colorectal cancer cell lines.

A. *KRAS* mRNA levels (N=72) relative to *DDX6* mRNA levels in COAD cell lines from CCLE database (Depmap portal). **B.** KRAS protein levels (N=28) relative to DDX6 protein levels in COAD cell lines. **C.** *KRAS* mRNA levels (N=72) relative to *EIF4ENF1* (4E-T) mRNA levels in COAD cell lines. **D.** NRAS protein levels (N=28) relative to EIF4ENF1 protein levels in COAD cell lines. **E.** *NRAS* mRNA levels (N=72) relative to *DDX6* mRNA levels in COAD cell lines. **F.** NRAS protein levels (N=28) relative to DDX6 protein levels in COAD cell lines.

Supp. Figure S5
