# Supplementary material for: KRAS and NRAS Translation Is Increased upon MEK Inhibitors-Induced Processing Bodies Dissolution
Source: Cancers (Basel). 2023 Jun 6;15(12):3078. doi: 10.3390/cancers15123078 (PMC10296394; doi:10.3390/cancers15123078)
Supplement: Supplementary file 1 [file cancers-15-03078-s001.zip › Figure S6.pdf]

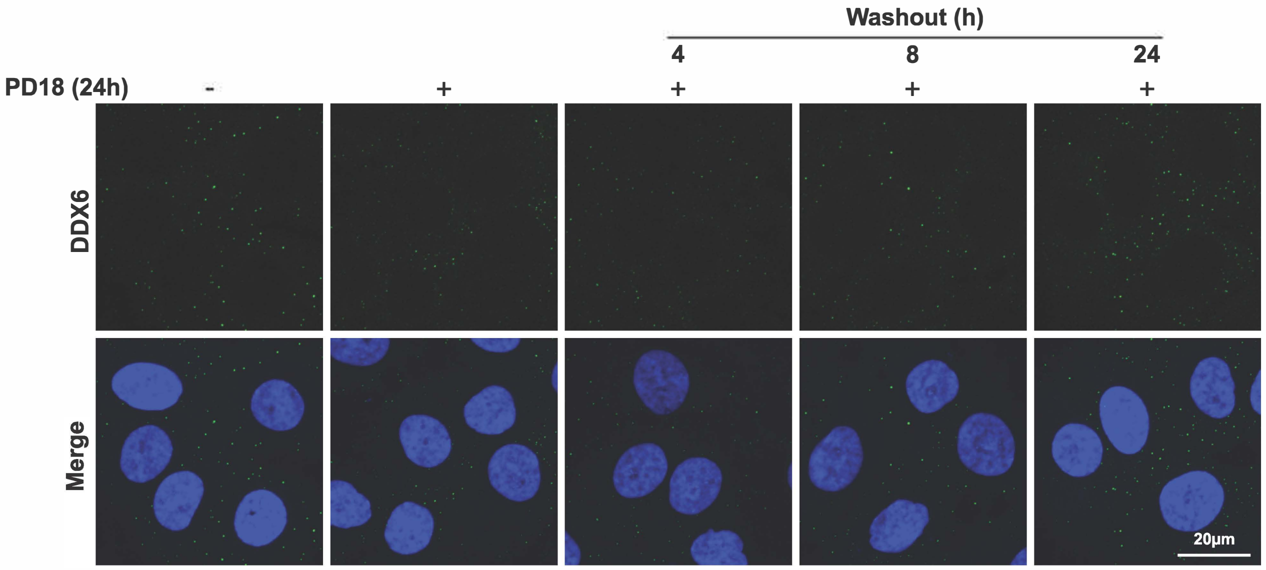

Supplementary Figure S6: Dynamic regulation of P-body formation.
A549 cells were treated with PD184352 (PD18) at 10μM, after 24h MEKi were washout, and cells were harvested at the indicated time. Confocal analysis of PBodies using anti-DDX6 (Green) antibodies with DAPI nuclear staining (Blue).

Supp. Figure S6
