# Supplementary material for: KRAS and NRAS Translation Is Increased upon MEK Inhibitors-Induced Processing Bodies Dissolution
Source: Cancers (Basel). 2023 Jun 6;15(12):3078. doi: 10.3390/cancers15123078 (PMC10296394; doi:10.3390/cancers15123078)
Supplement: Supplementary file 1 [file cancers-15-03078-s001.zip › Figure S7-13 .pdf]

Figure 1A

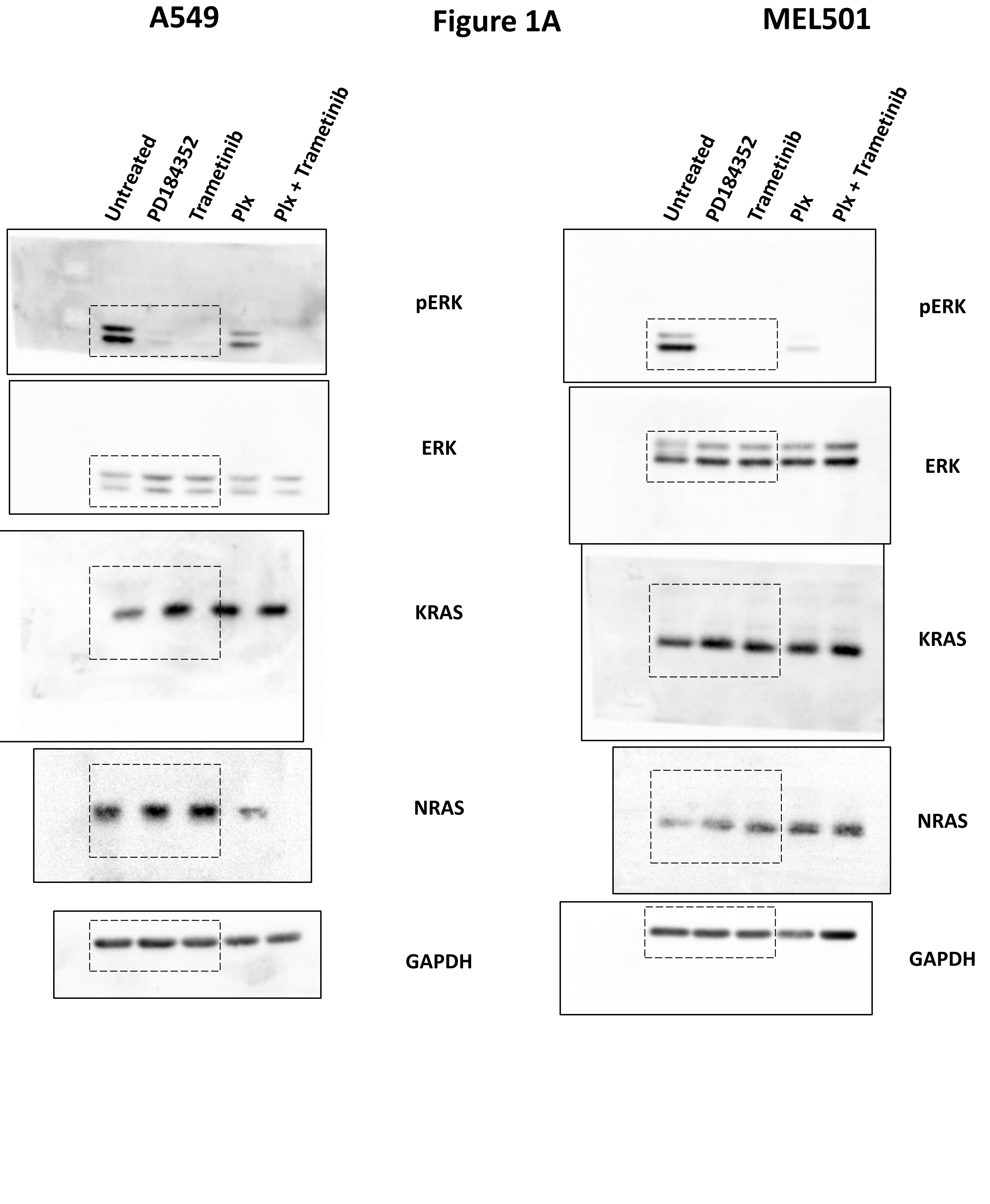

Suppl. Fig S7

BT549

Figure 1A

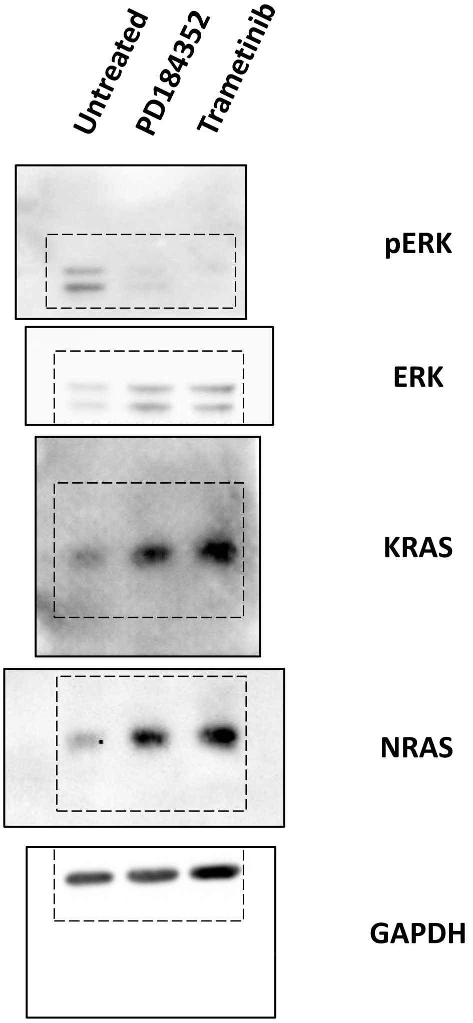

Suppl. Fig S8

Figure 3H

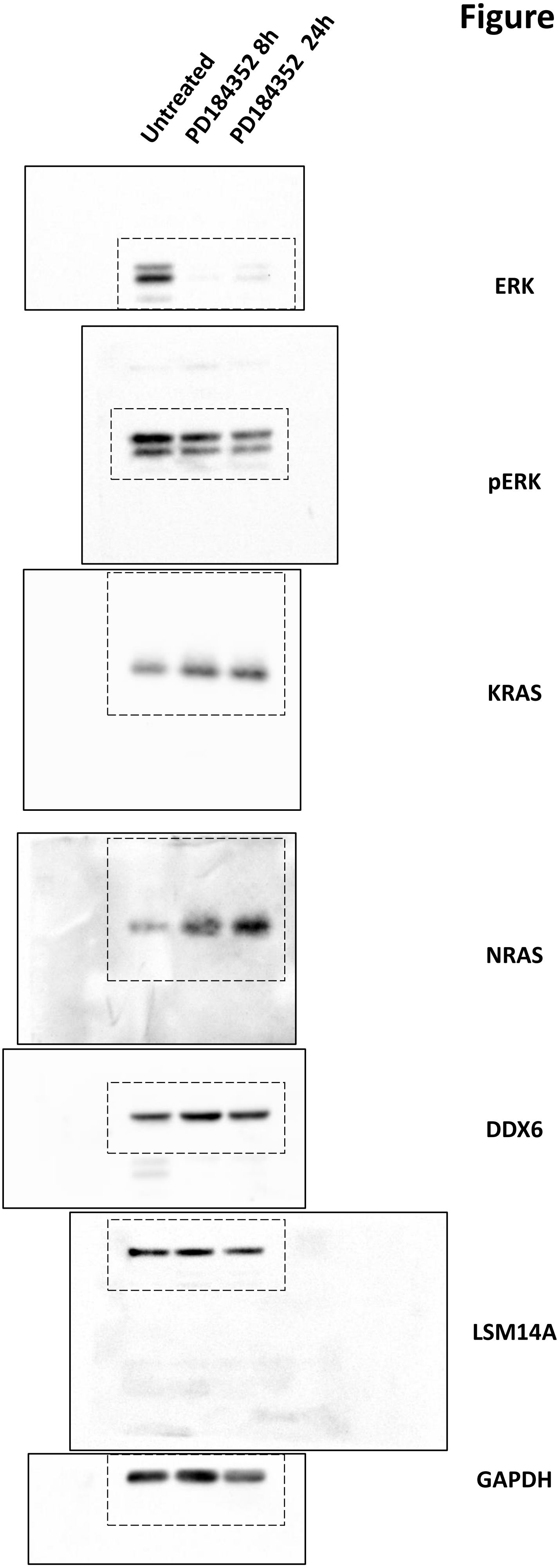

Suppl. Fig S9

Figure 4A

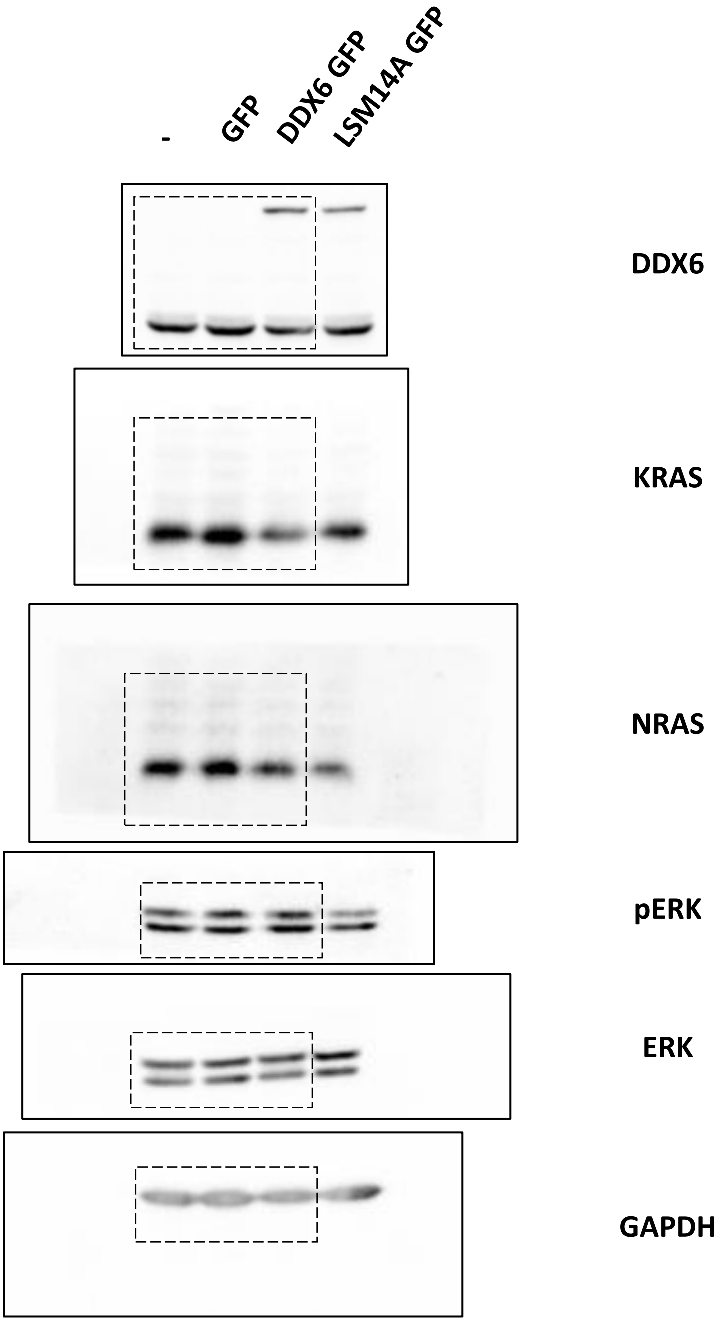

Suppl. Fig S10

Figure 5A

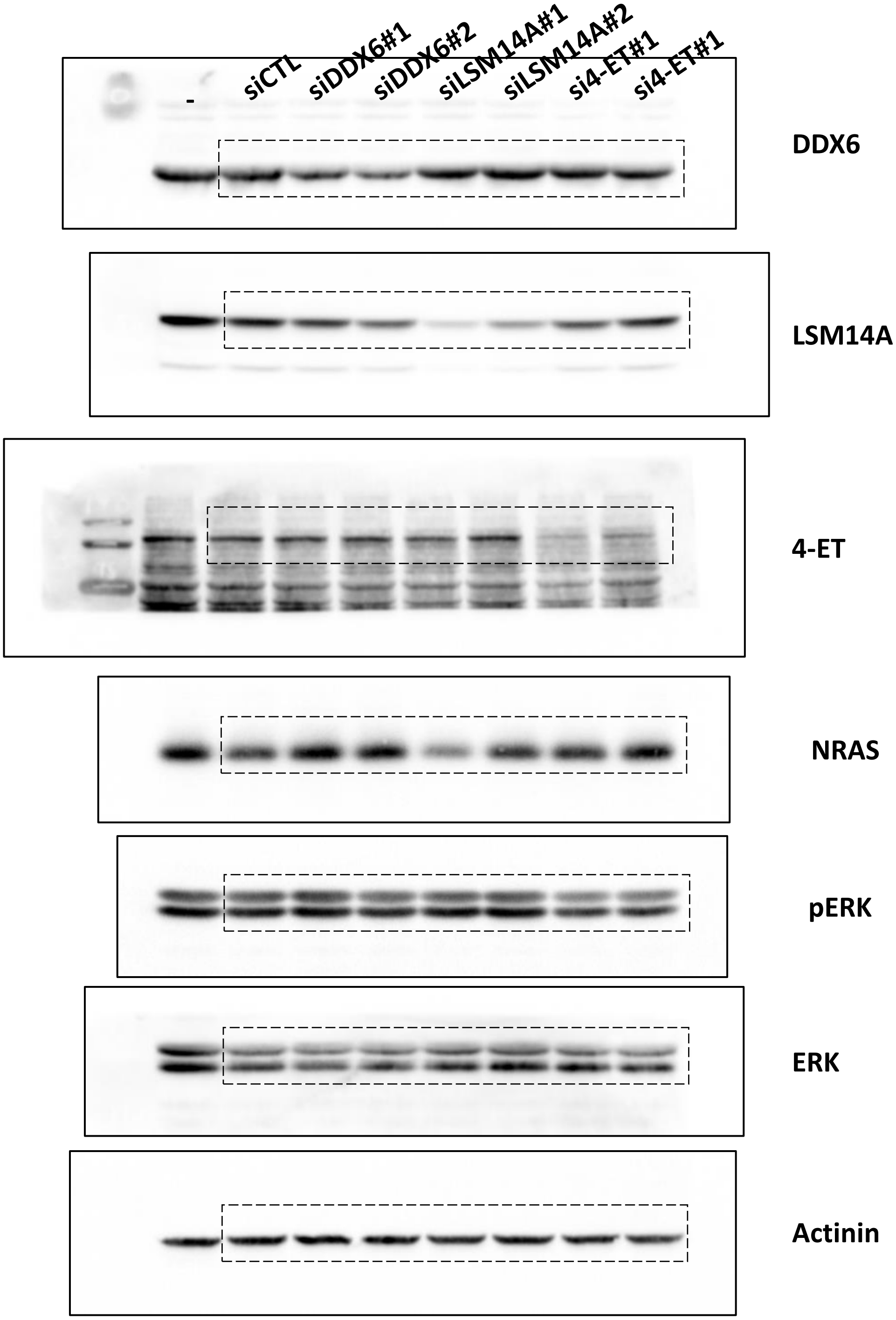

Suppl. Fig S11

Figure 7B

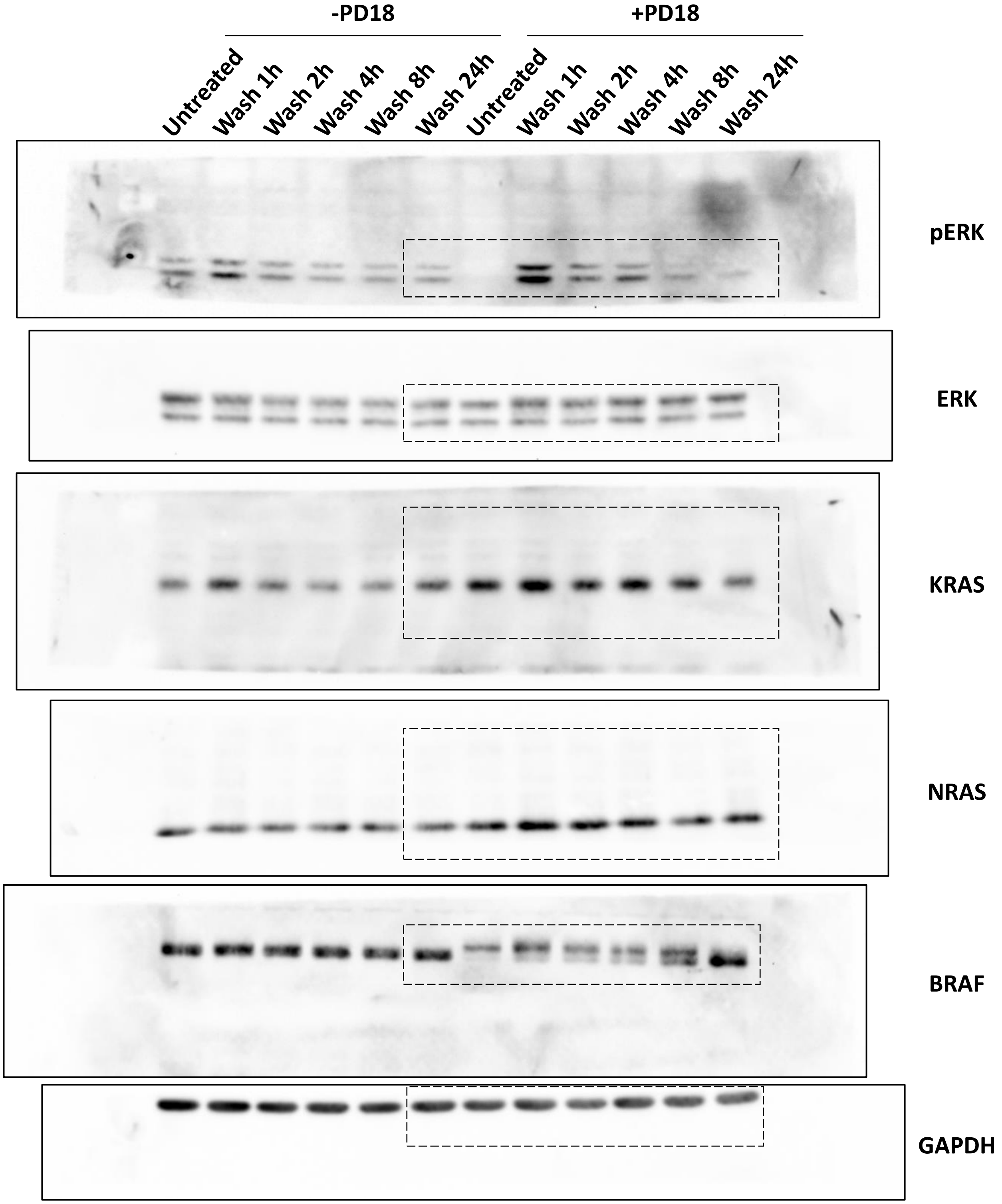

Suppl. Fig S12

Figure 8A

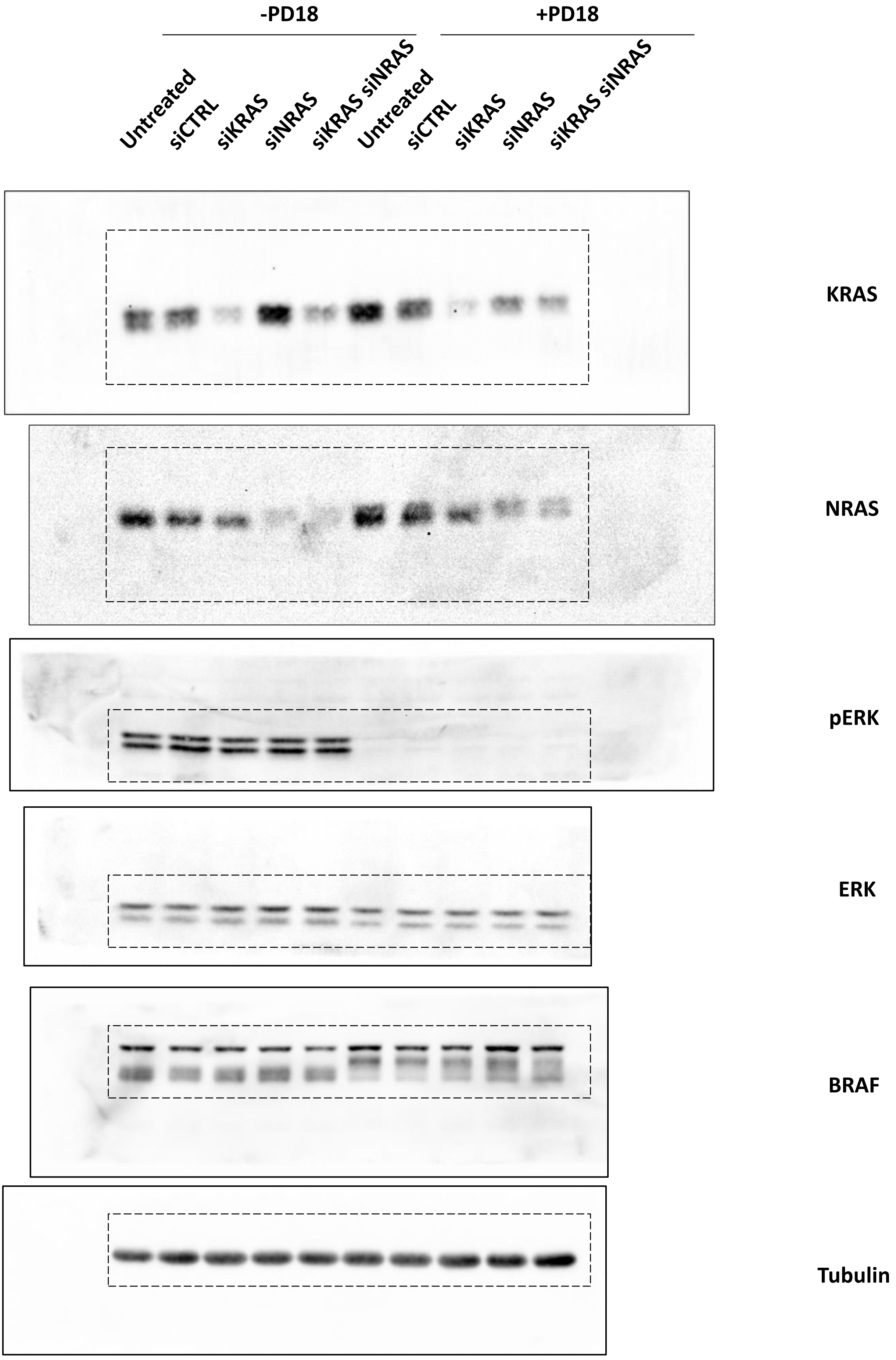

Suppl. Fig S13
