# Supplementary material for: KRAS and NRAS Translation Is Increased upon MEK Inhibitors-Induced Processing Bodies Dissolution
Source: Cancers (Basel). 2023 Jun 6;15(12):3078. doi: 10.3390/cancers15123078 (PMC10296394; doi:10.3390/cancers15123078)
Supplement: Supplementary file 1 [file cancers-15-03078-s001.zip › Table S1.pdf]

Supplementary Materials: KRAS and NRAS Translation Is Increased upon MEK Inhibitors-Induced Processing Bodies Dissolution

Olivia Vidal-Cruchez, Victoria J. Nicolini, Tifenn Rete, Karine Jacquet, Roger Rezzonico, Caroline Lacoux, Marie-Angela Domdom, Barnabé Roméo, Jérémie Roux, Arnaud Hubstenberger, Bernard Mari, Baharia Mograbi, Paul Hofman, and Patrick Brest

Table S1. List of reagents.

REAGENT or RESOURCE	SOURCE	IDENTIFIER
	**Antibodies**	
AlexaFluor 488 chicken anti-Rabbit IgG (IF:1/500)	Thermo Fisher Scientific Illkirch-Graffenstaden (Fr)	Cat# A-21441, RRID: AB_2535859
AlexaFluor 594 chicken anti-Rabbit IgG (IF:1/500)	Thermo Fisher Scientific	Cat# A-21442, RRID: AB_141840
Anti-mouse IgG (HRP conjugate) (WB:1/5.000)	Promega, Charbonnières-les-Bains (Fr)	Cat# W4021, RRID: AB_430834
Anti-rabbit (HRP conjugate) (WB:1/5.000)	Promega	Cat# W4011, RRID: AB_430833
Anti-TUBULIN (WB:1/30.000)	Sigma-Aldrich, Merck Millipore, Saint-Quentin-Fallavier (Fr)	Cat# T9026, RRID: AB_477593
Anti-BRAF (55C6) (WB:1/1.000)	Cell Signaling Technology, distributed by Ozyme, Saint-Cyr-l'École (Fr)	Cat# 9433, RRID: AB_2259354
Anti-DDX6 (WB:1/1.000) (IF:1/200)	Bethyl, distributed by Ozyme, Saint-Cyr-l'École (Fr)	Cat# A300-460A, RRID: AB_420926
Anti-DDX6 (IF:1/200)	Novus, distributed by Bio-Techne SAS, Noyal Châtillon sur Seiche (Fr)	Cat# NB 200-191, RRID: AB_523228
Anti-DDX6 (IF:1/200)	Santa Cruz Biotechnology, Heidelberg, (G)	Cat# sc-376433, RRID: AB_11151042
Anti-EIF4ENIF1 (WB:1/1.000)	Cell Signaling Technology	Cat# 2297, RRID: AB_2095735
Anti-G3BP1 (IF:1/200)	Santa Cruz Biotechnology	Cat# sc-365338, RRID: AB_10846950
Anti-GAPDH (WB:1/30.000)	Cell Signaling Technology	Cat# 2118, RRID: AB_561053
Anti-KRAS (WB:1/1.000) (IF:1/1000)	Novus	Cat# H00003845-M03, RRID: AB_1146050
Anti-LSM14A (WB:1/1.000) (IF:1/200)	Bethyl	Cat# A305-102A, RRID: AB_2631497
Anti-LSM14A (IF:1/500)	Santa Cruz Biotechnology	Cat# SC-398552
Anti-NRAS (F155) (WB:1/500) (IF:1/1000)	Santa Cruz Biotechnology	Cat# sc-31, RRID: AB_628041
Anti-p44/42 MAPK (ERK1/2) (WB:1/2.000)	Cell Signaling Technology	Cat# 9102, RRID: AB_330744
Anti-P-p44/42 MAPK (pERK) (WB:1/2.000)	Cell Signaling Technology	Cat# 9101, RRID: AB_331646
	**Chemicals, Peptides, and Recombinant Proteins**	
DC™ Protein Assay Kit	BioRad, Marnes La Coquette (Fr)	5000111
Direct-zol RNA MiniPrep Plus	ZymoResearch, distributed by Ozyme, Saint-Cyr-l'École (Fr)	R2072
Fast SYBR-Green Master Mix	Thermo Fisher Scientific	4385612
High-Capacity cDNA Reverse Transcription Kit	Thermo Fisher Scientific	4368814
Paraformaldehyde 32% solution EM grade	Electron Microscopy Science, distributed by Clinisciences, Nanterre (Fr)	15714
Prolong Diamond Antifade Mountant with DAPI	Thermo Fisher Scientific	P36962
Protein ladder	Euromedex, Souffelweyersheim (Fr)	06P-0111
RNAse OUT	Thermo Fisher Scientific	10777-019

TaqMan™ Fast Advanced Master Mix	Thermo Fisher Scientific	4444965
TaqMan™ MicroRNA Reverse Transcription Kit	Thermo Fisher Scientific	4366597
TRI Reagent®	Molecular Research Center, distributed by Euromedex.	TR 118
	**Cell culture and treatments**	
Puromycin Dihydrochloride	Thermo Fisher Scientific	A1113803
0,05% trypsin-EDTA (1X)	Thermo Fisher Scientific	25300-054
Amicon Ultra-15, PLHK, 100 kD	Sigma-Aldrich, Merck Milipore	UFC9100024
DDX6 silencer select #1	Thermo Fisher Scientific	4392420 (id : s4010)
DDX6 silencer select #2	Thermo Fisher Scientific	4392420 (id : s4012)
Dimethyl-sulfoxide	Sigma-Aldrich, Merck Milipore	276855-100mL
DMEM (1X) + Glutamax-I	Thermo Fisher Scientific	31966-021
EIF4ENIF (4E-T) silencer select #1	Thermo Fisher Scientific	4392420 (id : s32160)
EIF4ENIF (4E-T) silencer select #2	Thermo Fisher Scientific	4392420 (id : s32162)
Jet Prime transfection reagent	Polyplus, distributed by Ozyme	114-15
KRAS silencer select-validated	Thermo Fisher Scientific	4390824 (id : s7940)
Lipofectamine 3000	Thermo Fisher Scientific	L3000-008
LSM14A silencer select #1	Thermo Fisher Scientific	4392420 (id : s25051)
LSM14A silencer select #1	Thermo Fisher Scientific	4392420 (id : s25052)
MEM NEAA	Thermo Fisher Scientific	11140-35
Negative Control siRNA #1 Silencer Select	Thermo Fisher Scientific	4390843
NRAS silencer select-validated	Thermo Fisher Scientific	4390824 (id : s55)
PD184352	Sigma-Aldrich, Merck Milipore	P30181-5mg
Polybrene Infection / Transfection Reagent	Sigma-Aldrich, Merck Milipore	TR-1003-G
Sodium Arsenite Solution	Sigma-Aldrich, Merck Milipore	106277
Trametinib	TargetMOI, distributed by Tebu-Bio, Le Perray-en-Yvelines (Fr)	TA-T2125-5mg
	**Oligonucleotides**	
KRAS-Forward	Eurofins Genomics, Ebersberg (G)	GACTGGGGAGGGCTTTCTTT
KRAS-Reverse	Eurofins Genomics	GCATCATCAACACCCTGTCT
NRAS-Forward	Eurofins Genomics	CCAATACATGAGGACAGGCGA
NRAS-Reverse	Eurofins Genomics	TCACACTTGTTTCCCACTAGCA
RPLP0-Forward	Eurofins Genomics	GCATCAGTACCCCATTCTATCAT
RPLP0-Reverse	Eurofins Genomics	AGGTGTAATCCGTCTCCACAGA
	**Recombinant DNA**	
pEGFP-C1_p54cp	Gift	
pPRIPu-CrUCCI	Gift	
pPRIPu-pEGFP-C1_p54cp	In house	
	**Software and Algorithms**	
CytExpert 2.3	Beckman Coulter,Villepinte (Fr)	
FIJI 1.53j		RRID:SCR_002285
GraphPad Prism version 8.0.2 for Windows,	GraphPad Software, La Jolla (USA)	RRID:SCR_002798
StepOne Software v2.3	Thermo Fisher Scientific	
